# Machine Learning of Motion Statistics Reveals the Kinematic Signature of the Identity of a Person in Sign Language

**DOI:** 10.3389/fbioe.2021.710132

**Published:** 2021-07-22

**Authors:** Félix Bigand, Elise Prigent, Bastien Berret, Annelies Braffort

**Affiliations:** ^1^CNRS, LISN, Université Paris-Saclay, Orsay, France; ^2^CIAMS, Université Paris-Saclay, Institut Universitaire de France, Orsay, France

**Keywords:** person identification, human movements, feature extraction, motion capture, machine learning, statistics, sign language

## Abstract

Sign language (SL) motion contains information about the identity of a signer, as does voice for a speaker or gait for a walker. However, how such information is encoded in the movements of a person remains unclear. In the present study, a machine learning model was trained to extract the motion features allowing for the automatic identification of signers. A motion capture (mocap) system recorded six signers during the spontaneous production of French Sign Language (LSF) discourses. A principal component analysis (PCA) was applied to time-averaged statistics of the mocap data. A linear classifier then managed to identify the signers from a reduced set of principal components (PCs). The performance of the model was not affected when information about the size and shape of the signers were normalized. Posture normalization decreased the performance of the model, which nevertheless remained over five times superior to chance level. These findings demonstrate that the identity of a signer can be characterized by specific statistics of kinematic features, beyond information related to size, shape, and posture. This is a first step toward determining the motion descriptors necessary to account for the human ability to identify signers.

## Introduction

Sign languages (SLs) are the natural languages used in Deaf communities. SL users express themselves by producing a continuous stream of movements with numerous body parts, such as hands and arms and eye gaze, facial expressions, and torso. With the advent of motion capture (mocap) systems, it has been possible to develop virtual signers (or signing avatars) with high naturality and comprehensibility, by replaying movements of real signers (Lu and Huenerfauth, 2010, 2014; Gibet, 2018). However, it has now been shown that deaf observers can identify signers from point-light displays (PLDs) of their movements, beyond cues related to appearance, clothes, or morphology (Bigand et al., 2020). This observation questions the possibility to produce anonymized, non-identifiable, content with virtual signers. Compared to the auditory domain where a speaker can remain anonymous by modifying specific voice characteristics, little is known about the motion features that characterize the identity of a signer. This problem is crucial given that SLs have no written form. For deaf persons whose first language is a sign language, reading written content means reading a second language, which is not always mastered (Holt, 1993). Professional journals that provides accessible content in SL, such as Media'Pi! in France ^1^, have raised the need for novel technological tools to allow signers to remain anonymous when expressing themselves (e.g., for sharing anonymized testimony). For all these reasons, technological barriers must thus in order to provide.

Visual perception studies were the first to provide evidence that human motion conveys important information about the moving person, using PLDs. PLDs isolate information given by motion cues from information given by other characteristics, such as shape or other aspects of the body of a person (Johansson, 1973). Using PLDs, studies have demonstrated that human observers were able to extract critical information from motion, such as actions (Johansson, 1973), gender (Kozlowski and Cutting, 1977; Mather and Murdoch, 1994), or emotional state (Atkinson et al., 2004). Similarly, behavioral studies have used PLDs to show that the identity of familiar individuals can be inferred from human movements, such as walking (Cutting and Kozlowski, 1977; Loula et al., 2005; Troje et al., 2005), dancing (Loula et al., 2005; Bläsing and Sauzet, 2018), clapping (Sevdalis and Keller, 2009), or producing SL (Bigand et al., 2020). Moreover, Baragchizadeh et al. (2020) recently demonstrated that motion cues also allow for the perceptual discrimination of the identity of unfamiliar people.

Beyond the overall ability of humans to infer identity from motion, only a few perceptual studies have aimed to determine the cues that allow for the identification. According to Troje et al. (2005), removing the size of walkers and shape information from PLDs had only a low impact on human identification accuracy, which was still five to six times above chance level. These results suggest that most of the information used for identification is conveyed by motion kinematics. The nature of such kinematic cues remains relatively unclear up to now. According to Troje et al. (2005) and Westhoff and Troje (2007), gait frequency may not play a major role in identification. The most critical information for identification seems to be conveyed by the first harmonic and the amplitude spectrum of walking patterns (Westhoff and Troje, 2007). In addition to human perception measurements, other approaches, such as machine learning, can provide further insights along this line. Some machine learning models have been successfully trained to identify walkers (Zhang and Troje, 2005) and dancers (Carlson et al., 2020) from mocap data. Both studies concluded that most of the critical information for identity was conveyed by motion kinematics, as for the automatic gender classification of gait (Troje, 2002). Further investigation is needed to better understand the role of kinematic cues in the perception of the identity of an individual, in particular for SL. One specific aspect of SL is to be governed not only by biomechanic rules, but also by linguistic ones, which may thus reveal SL-specific signatures for the identity of signers.

In American and Croatian Sign Language, Malaia and Wilbur (2012) and Malaia et al. (2013) have demonstrated that kinematic features (e.g., peak speed, instantaneous acceleration) of verb signs were affected both by predicate type (telic/atelic) and the position of the sign within the sentence (medial/final). This suggests that kinematics may convey relevant information about both semantics and prosody. In French Sign Language (LSF), Catteau et al. (2016) have outlined kinematic strategies of interpreters (e.g., acceleration peaks of the whole-body joints) to convey prosodic variation. The study of LSF mocap from elderly signers has also suggested that specific kinematics, such as signing rate, may provide a prosodic characterization for the age of a signer (Blondel et al., 2019). However, up to now, neither approaches using perceptual measures nor machine learning methods have attempted to determine the parameters of SL motion that convey the identity of a signer.

Identity is a time-invariant property that humans can recognize from different utterances of the same individual. This makes time-averaged statistics a particularly suited description to extract identity-specific features. In the auditory domain, Latinus and Belin (2011) have shown that the dissimilarities of speakers, across brief vowel utterances, were well explained using the average fundamental frequency of phonation (F0) and the average first formant frequency (F1). The role of statistics for categorical discrimination of sounds has been shown with human behavioral data in McDermott et al. (2013), revealing that discrimination of sounds improved with longer excerpts, notably for the recognition of a single speaker. Converging evidence has been provided by machine learning of human motion: a linear regression model trained by Tits (2018) has been able to accurately predict the level of expertise from gesture in Taijiquan, based on the mean and SD of position and velocity. Moreover, Carlson et al. (2020) recently demonstrated that the identity of a dancer may be encoded by the covariance of three-dimensional movements between specific body markers.

The present study aimed to determine the information that allows for identifying signers in LSF. We used machine learning to determine the parts of motion information that are responsible for the identification. For that aim, we (1) evaluated to what extent a machine learning model managed to identify six different signers from statistics of mocap utterances in spontaneous LSF; (2) assessed the distinct roles of structural and kinematic information in the model identification, by gradually normalizing the mocap data according to size, shape, and posture of the signers; (3) further examined the identity-specific features extracted by the model when trained on posture-normalized mocap data.

## Materials and Methods

### Motion Capture Corpus

**Figure 1:**
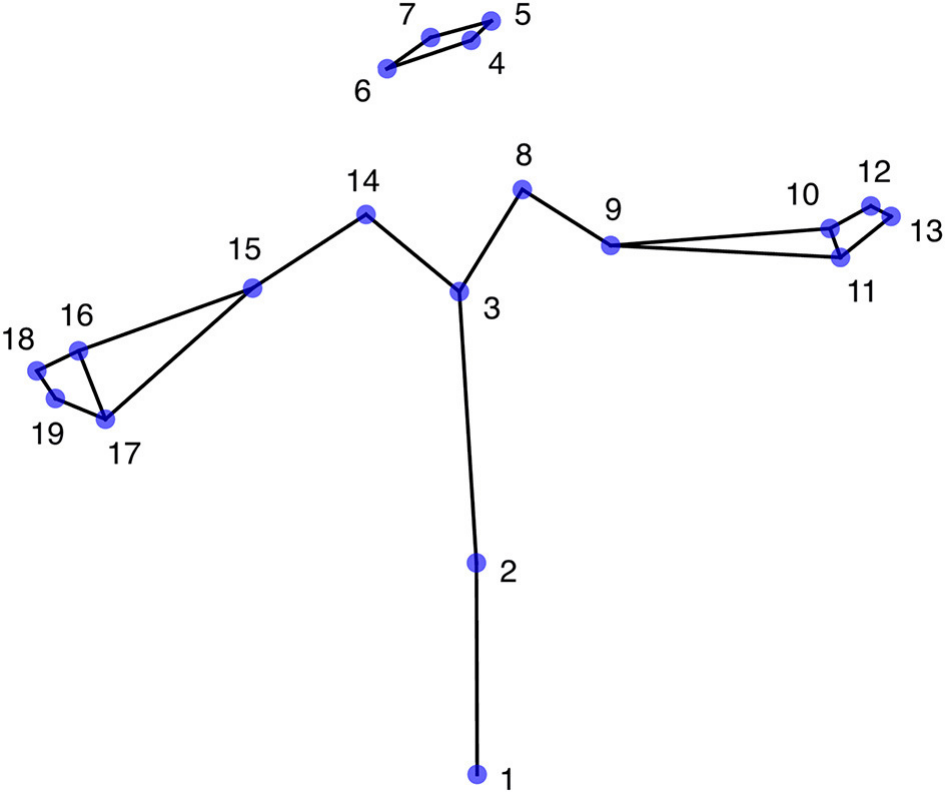
The 19 upper-body markers in the “T” reference posture.

The data used in the present investigation were taken from a previously reported study (Bigand et al., 2020). In brief, each of six deaf native and fluent signers had freely described the content of 25 pictures (as shown in examples in Supplementary Material 1) using LSF. The dominant hand of all signers was the right hand. Using a mocap system equipped with 10 cameras (Optitrack S250e), the data consisted of the upper-body movements recorded at 250 fps in three dimensions. Further details (e.g., picture content, type of SL discourse, and mocap equipment) are available from the original mocap corpus (Benchiheub et al., 2016a,b). From the 25 mocap recordings, only 24 were taken into account in the present study, as one of them was not available for one signer. Moreover, from the 27 original body markers, we derived 19 virtual markers that optimally describe the major joints of the body. As shown in Figure 1, the derived markers were (L = left, R = right, F = front, B = back): (1) pelvis, (2) stomach, (3) sternum, (4) LB head, (5) LF head, (6) RB head, (7) RF head, (8) L shoulder, (9) L elbow, (10) LB wrist, (11) LF wrist, (12) LB hand, (13) LF hand, (14) R shoulder, (15) R elbow, (16) RB wrist, (17) RF wrist, (18) RB hand, (19) RF hand.

The positions of all markers were defined in reference to the pelvis (used as the origin) and low-pass filtered using a fourth-order Butterworth filter with a cutoff frequency of 12 Hz, following recent estimations of SL kinematic bandwidth (Bigand et al., 2021). From each of the 24 original recordings, one mocap recording unit with a duration of 5 s was extracted from the beginning of the utterance, irrespective of the semantic content. Each mocap recording unit was thus related to a different SL utterance. This resulted in 24 mocap examples per signer, of 5 s duration each (as shown in examples in Supplementary Materials 2–7).

### Normalizations of Structural Features

Two classes of information can be distinguished when studying the perception of movements: structural and kinematic information. Motion-mediated structural features were defined by Troje et al. (2005) as the invariant information specifying the structure of the body that is put into motion. For instance, structural features reveal information about the average posture, and the anthropometric characteristics of the body of a person. For structural features to be perceived, PLDs must be in motion. Motion-mediated structural features thus differ from static information, which can be perceived from a static PLD image. However, although they are inferred from moving PLDs, structural features also differ from kinematic ones, which refer to the motion of the body markers themselves.

**Figure 2:**
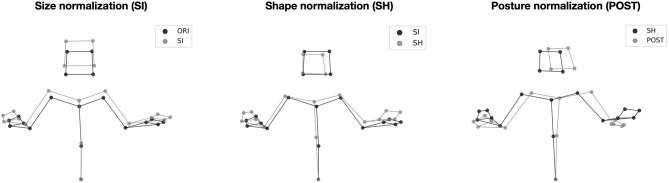
The three cumulative steps of normalizations of structural features. The stick figures correspond to a given frame of the description of the first picture by Signer 3. For each step, the normalized and non-normalized stick figures are compared.

To evaluate the distinct roles of structural and kinematic information in identification, the original mocap data (referred to as “ORI”) used in the present study were gradually normalized in three steps (illustrated in Figure 2), with respect to size (SI), shape (SH), and posture (POST) of the signers, respectively. The shape was defined as the individual lengths of the body segments of a signer, such as shoulder width, arm length, or dimensions of the head. Posture was defined as the average position of the body markers of a signer (i.e., how the signer holds his or her body) over all mocap examples. Compared to the two-step normalization procedure proposed by Troje et al. (2005), the three normalization steps proposed here allowed us to distinguish the role of postural information from the one of size and shape, which latter are related to the dimensions of the body of an individual, regardless of his or her average posture.

- Size normalization (SI): Reference “T” postures were recorded for each signer (Figure 1). An overall reference “T” posture was computed by averaging across the six signers. The slope of the regression between each reference posture and the overall reference posture was then computed. These slopes defined relative sizes (Troje et al., 2005) for each signer: 1.000, 1.075, 0.924, 1.003, 0.996, and 1.003. After dividing the position coordinates of the signer by their sizes, they all had the same size. This normalization kept intact shape (i.e., the relative positions of the articulations).
- Shape normalization (SH): New reference “T” postures were computed from the size-normalized data of each signer. New overall reference “T” posture was defined. Shape-normalized data were obtained by subtracting individual reference postures from each frame, then adding the overall reference posture. After that transformation, all signers had the same reference “T” posture (i.e., same relative positions of the articulations).
- Posture normalization (POST): This last normalization was applied to shape-normalized data, which are also size-normalized. Posture-normalized data were obtained by subtracting the average posture of each signer (averaged over all their mocap examples) from each frame, then adding the average posture computed over all signers. After these three normalizations, all signers had the same size, same shape, and same average posture.

### Feature Extraction: A Statistical-Based Approach

**Figure 3:**
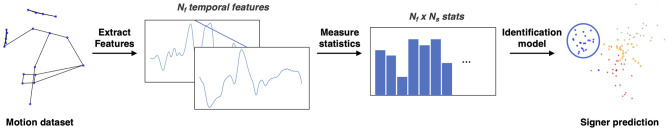
Schematic representation of the steps used in the machine learning model for identification.

The machine learning workflow is displayed in Figure 3. The mocap data of the pelvis marker were ignored as it was set as the origin, which leads to zero vectors. The position and velocity of the 18 other markers were used as temporal features. Velocity was estimated by time differentiation of the mocap position coordinates (ORI, SI, SH, or POST). Then, we measured the statistics of these temporal features.

Based on previous research investigating the perception of auditory and visual textures (Portilla and Simoncelli, 2000; McDermott and Simoncelli, 2011), we measured the first four moments of position and velocity (Equation 1), and covariances of velocity between body markers (Equation 2). The first four moments of position and velocity described their statistical distributions, which may vary from one individual to another, as shown for expert gesture analysis (Tits, 2018). For instance, for the position, the mean provides information about the average posture of the signers, and the SD provides information about the amplitude of their movements. For velocity, SD provides information about the amount of velocity the markers of a signer in any of the three dimensions. Although the interpretation of the other moments is more challenging, their role in the identification was tested, similarly to McDermott and Simoncelli (2011). Moreover, the covariance of velocity allowed for quantifying the extent to which any two markers covaried with each other, in two directions. This latter statistic has been shown to allow for automatic person identification from dance movements (Carlson et al., 2020).

For each mocap example, the triangular part of the covariance matrix was reshaped into a vector of length 1,431 and concatenated with the moments of position and velocity, of length 53 each. The concatenated statistics constituted the feature vector used in the person identification model. By definition, posture-normalized data had the same mean position so this latter statistic was not included in the POST condition. The computation of the first four moments (Equation 1) and covariance (Equation 2) is detailed as follows:

(1) $$\begin{array}{l} M_{1,k}=\;\mu_{k}=\frac{1}{T}\overset{T}{\underset{t=1}{\sum }}x_{k}\left(t\right),\\ M_{2,k}=\sigma_{k}=\sqrt{\frac{1}{T}\overset{T}{\underset{t=1}{\sum }}{\left(x_{k}\left(t\right)-\mu_{k}\right)}^{2}},\\ M_{3,k}=\;\frac{\frac{1}{T}\sum_{t=1}^{T}{\left(x_{k}\left(t\right)-\mu_{k}\right)}^{3}}{\sigma_{k}^{3}},\\ M_{4,k}=\;\frac{\frac{1}{T}\sum_{t=1}^{T}{\left(x_{k}\left(t\right)-\mu_{k}\right)}^{4}}{\sigma_{k}^{4}}-3 \end{array}$$

*x*_*k*_ is the temporal feature (position or velocity) of a marker, along one of the three directions. *k* ∈ [1, 54].

(2) $$\begin{array}{l} C_{i,j}=\frac{1}{T-1}\overset{T}{\underset{t=1}{\sum }}\left(x_{i}-\mu_{i}\right)\left(x_{j}-\mu_{j}\right) \end{array}$$

*x*_*i,j*_ are temporal features related to two markers. μ_*i,j*_ is the mean of the feature. *i, j* ∈ [1, 54].

### Person Identification

To predict the identity of the signer, principal component analysis (PCA) followed by a classifier was used. PCA was applied to the motion statistics (contained in a matrix either of length 144 × 1,863 for ORI, SI, and SH; or 144 × 1,809 for POST) and provided uncorrelated principal components (PCs) (or eigenvectors), which are linear combinations of the original statistics:

(3) $$\begin{array}{l} \text{D}={\text{d}}_{0}+\text{X}\text{V} \end{array}$$

The matrix **D** contains the original statistics of all examples, vector **d**_**0**_ contains the average statistics across examples, matrix **X** contains the coefficients of the original statistics of all examples in the PC space and matrix **V** contains the PCs (or eigenvectors).

This data-driven method allowed extracting candidate components for the characterization of identity, without a priori hypotheses on the statistics. It also allowed for dimensionality reduction, enabling us to retain a reduced number of PCs. The number of retained PCs has often been chosen based on the amount of variance they explained (Zago et al., 2017a). In the present study, the number of selected PCs was chosen so that it maximized identification accuracy, by testing the model with an increasing number of PCs (based on the descending order of the variance they explained). This follows the approach proposed by O'Toole et al. (1993) who have shown that for face identification, higher-order PCs, which explain only few variances, capture identity-specific features while most of the variance is covered by low-order PCs.

On the reduced set of PCs, a classifier was trained. We have tested the differences in performance between different classifiers, notably between linear and non-linear ones. As a model comparison is beyond the scope of the study, we present the classifier that reported the highest performance, that is multinomial logistic regression ^2^. For the prediction of each signer, a logistic regression model was trained, as defined in Equation 4.

(4) $$\begin{array}{l} P\left(S=s\right)=\frac{e^{\beta_{\text{s}}.\text{X}}}{\overset{6}{\underset{k=1}{\sum }}e^{\beta_{\text{k}}.\text{X}}} \end{array}$$

**X** is the vector containing the coefficients of the test data in the PC space, the vector *β*_**k**_ contains the regression coefficients optimized for the identification of signer *k* during the learning step, and *S* is the signer variable. The signer *s* reaching the highest probability in the model is defined as the predicted signer.

A leave-one-out cross-validation was conducted: the model was trained on N-1 (23) mocap examples for each signer, and the remaining mocap example was used as the test example (i.e., an unknown example that the model must identify as the production of the signer). All examples were used as test example so the model was tested 24 times and performance was computed as an average across these iterations. Using this cross-validation step, we assessed to what extent the classifier learned idiosyncratic movement statistics that generalize to new mocap examples.

Finally, to better understand the motion statistics that allowed for identification, we scrutinized some discriminant PCs (i.e., PCs that contributed to a significant increase in identification accuracy) in terms of the original statistics they described. First, the general statistical patterns **d**_**n**_ of each PC were described as the absolute value of the PC (**V**_**n**_) (Equation 5). Based on these descriptions, we then proposed some interpretation of the motion information these PCs might contain. Second, the optimized regression coefficient that the classifier assigned to a given PC for the identification of Signer *k* was projected onto the PC (Equation 6). The resulting statistical patterns **d**_**n,k**_ provided further insights about the differences between signers along the given PC (**V**_**n**_).

(5) $$\begin{array}{l} {\text{d}}_{\text{n}}=|{\text{V}}_{\text{n}}| \end{array}$$

(6) $$\begin{array}{l} {\text{d}}_{\text{n},\text{k}}=\beta_{n,k}{\text{V}}_{\text{n}} \end{array}$$

**V**_**n**_ is the *n*^*th*^ PC (or eigenvector) of the PC space, the scalar β_*n,k*_ is the optimized regression weight assigned to **V**_**n**_ by the classifier to identify signer *k*. **d**_**n**_ and **d**_**n,k**_ are vectors containing the statistical patterns (e.g., of length 1,809, in POST condition).

## Results

### The Role of Structural and Kinematic Features

**Figure 4:**
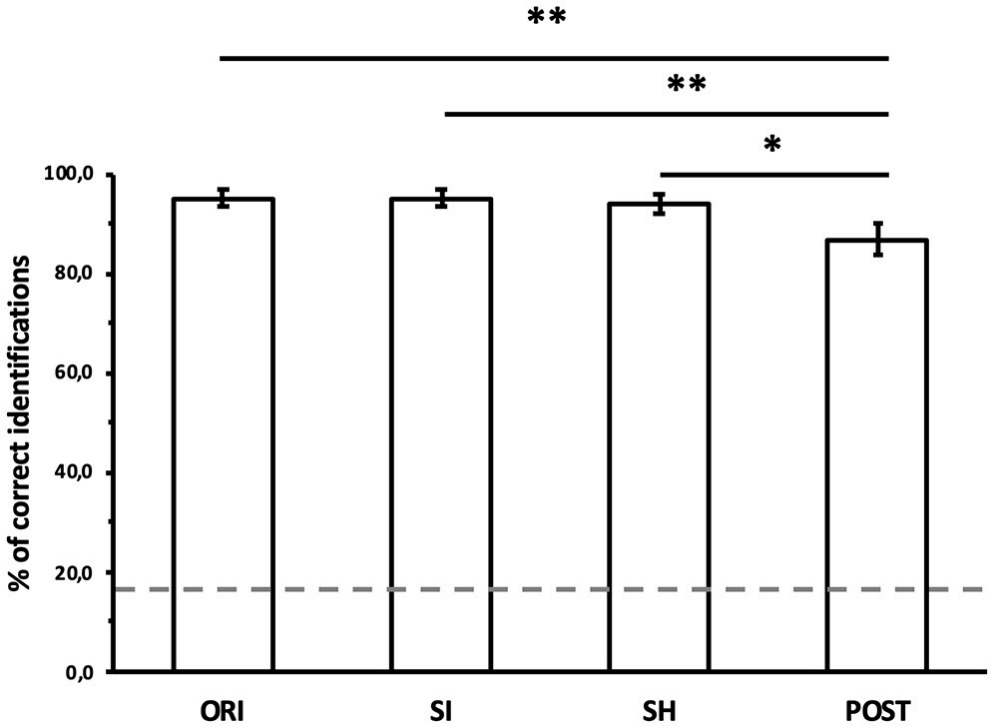
Average correct identifications of the model, as a function of the normalizations of structural features. ORI, original motion; SI, size-normalized; SH, shape-normalized; POST, posture-normalized. Dashed horizontal line indicates the chance level. Error bars indicate SE. Significant differences between normalizations: *p < 0.05, **p < 0.01.

Correct identifications of the model as a function of the normalizations are shown in Figure 4. A repeated measures one-way ANOVA with normalization (with its four levels: ORI, SI, SH, and POST) as within-test factor was run on correct identifications. As the assumption of sphericity was violated (Mauchly's test, *p* < 0.05), a Greenhouse-Geisser correction was applied (ε = 0.61). The main effect of normalization was significant [$F_{\left(1.83,42.07\right)}=5.46,p<0.01,\eta^{2}=0.19$]. Bonferroni-adjusted *post-hoc* tests were performed to test for differences between normalizations. They revealed a significant increase of identification accuracy from POST (mean = 86.8%) to SH (mean = 93.8%, *p* < 0.05), SI (mean = 95.1%, *p* < 0.01) and original motion (ORI, mean = 95.1%, *p* < 0.01). No significant difference was found between original, size normalization, or shape normalization (*p* > 0.05).

### The Identification Accuracy of the Model for Posture-Normalized Motion

**Figure 5:**
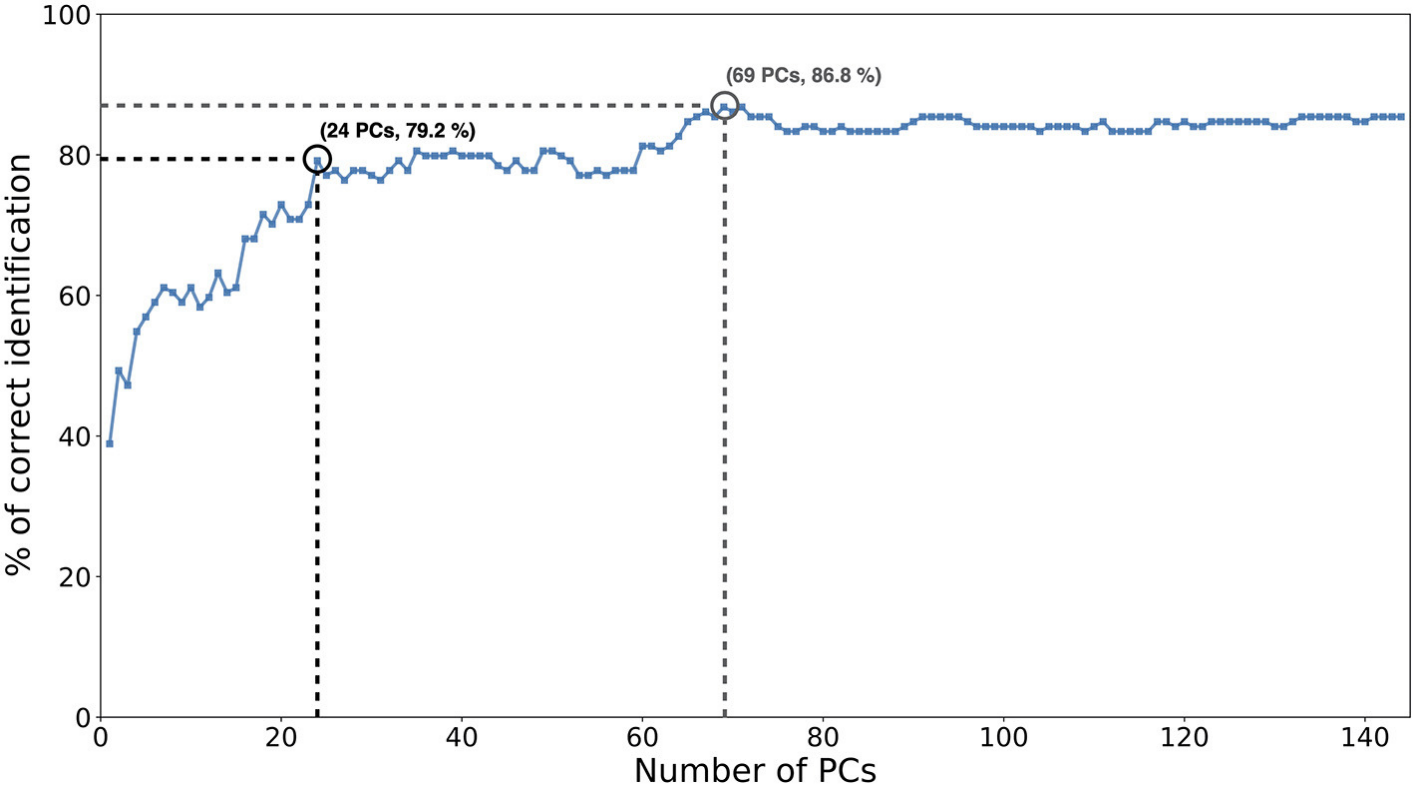
Correct identifications of the model from POST motion, as a function of the number of principal components (PCs), used. The first 24 PCs contributed to most of the correct identifications of the model (79.2%). The highest accuracy (86.8%) was obtained with 69 PCs.

Figure 5 displays the correct identifications of the model when trained on POST mocap data. The number of retained PCs varied from 1 to 144 (which corresponds to the number of mocap examples across signers) (for further details about how the PCs were retained, see Methods). The highest accuracy of 86.8% was obtained using 69 components. The first component alone allowed for a 38.9% average correct identification. The first 24 components alone contributed to most of the correct identifications, with a 79.2% accuracy. Components 59–69 then contributed to most of the increase toward the highest accuracy, from 77.8 to 86.8%.

Table 1 presents the confusion matrix of the model trained with 69 components, which leads to the highest identification accuracy. It specifies the predictions for each signer, across the 24 examples. One sample Student's *t*-tests revealed that identification performance was above chance level (16.7%) for all signers [*p* < 0.001, Signer 1: *t*_(23)_ = 9.33, *d* = 1.91, Signer 2: *t*_(23)_ = 10.27, *d* = 2.59, Signer 3: *t*_(23)_ = 18.99, *d* = 4.69, Signer 4: *t*_(23)_ = 7.47, *d* = 1.53, Signer 5: *t*_(23)_ = 8.58, *d* = 2.19, Signer 6: *t*_(23)_ = 18.99, *d* = 4.69]. No confusions were significant between signers (*p* > 0.05). The lowest performance of the model occurred for Signer 4, with a 70.8% accuracy.

**Table 1:** Confusion matrix displays the percentage of identifications of the model, averaged across the 24 test examples (for posture-normalized motion).

	**Signer 1**	**Signer 2**	**Signer 3**	**Signer 4**	**Signer 5**	**Signer 6**
Signer 1	79.2^***^	0	0	8.3	12.5	0
Signer 2	0	87.5^***^	4.2	4.2	0	4.2
Signer 3	0	4.2	95.8^***^	0	0	0
Signer 4	8.3	8.3	4.2	70.8^***^	4.2	4.2
Signer 5	16.7	0	0	0	83.3^***^	0
Signer 6	4.2	0	0	0	0	95.8^***^

*Accuracy values significantly above chance level are shown in bold:*

*** *(p < 0.001)*.

### Kinematic Features of Importance

**Figure 6:**
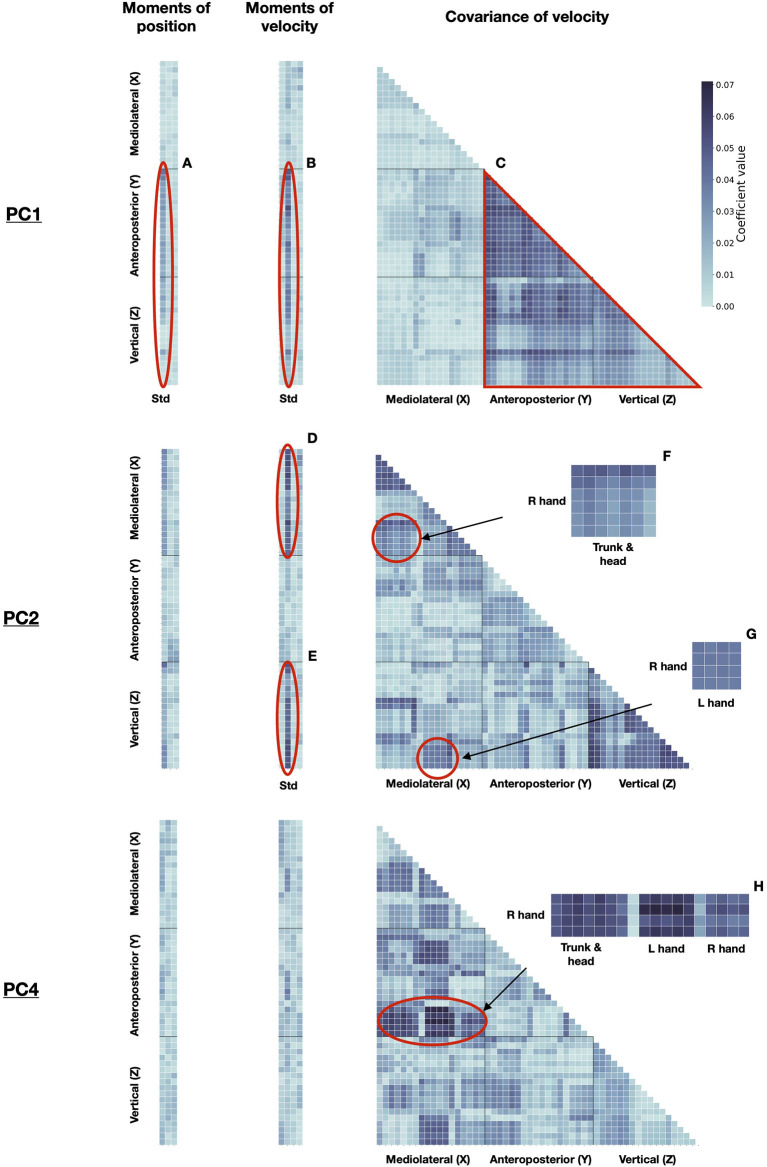
Discriminant PCs for signer identification. Left: moments (columns: std, skew, kurtosis) of position, for all markers (rows). Middle: moments (columns: mean, std, skew, kurtosis) of velocity, for all markers (rows). Right: covariance of velocity between markers (rows and columns). Markers are sorted from 1 to 19 as presented in the Methods along X, Y, and Z axes. Some patterns of importance are highlighted. For the sake of clarity, the specific moments and body markers are displayed only for these patterns of importance. PC1: Std of position **(A)** and velocity **(B)** along Y and Z axes, for all markers; **(C)** Covarying movements between all markers along Y and Z axes. PC2: Std of velocity along X **(D)** and Z **(E)** axes, for all markers; **(F)** Covarying movements between the right hand, and trunk, and head markers, along *X*-axis; **(G)** Covarying movements between the right hand markers along *Z*-axis, and the left hand markers along *X*-axis. PC4: **(H)** Covarying movements between the right hand markers along the *Y*-axis, and all other markers along the *X*-axis.

To further understand which kind of information is useful for signer identification from POST motion, we examined the PCs used by the classifier. The identification model was run on the whole dataset with the 69 components, which allowed reaching the highest performance. Discriminant PCs were described following equation 5 (as shown in Methods). The statistical patterns (referred to as **d**_**n**_ in Equation 5) of some highly discriminant PCs are displayed in Figure 6. PC1, PC2, and PC4 contributed to 38.9%, 10.4 and 7.6% of the cumulative correct identification, respectively (Figure 5).

Principal component 1 mainly described relationships between movements along vertical (Z) and anteroposterior (Y) axes, except between hand markers along the *Z*-axis and head markers along the *Y*-axis (Figure 6C). It also described differences in standard deviations of the position and velocity for all body joints along the *Y*-axis (Figure 6A), and for the trunk and head along the *Z*-axis (Figure 6B). PC2 was mostly related to movements along the mediolateral (X) (Figure 6D) and Z-axes (Figure 6E). Covarying movements of the head with the right hand along the *X*-axis (Figure 6F) are characteristic of this PC and the right hand with the left hand along Z and X axes, respectively (Figure 6G). PC4 did not describe global movements along with one of the three axes, compared with PC1 and PC2. Instead, it mainly characterized relationships between movements along the X and Y axes, particularly regarding the right hand (Figure 6H).

**Figure 7:**
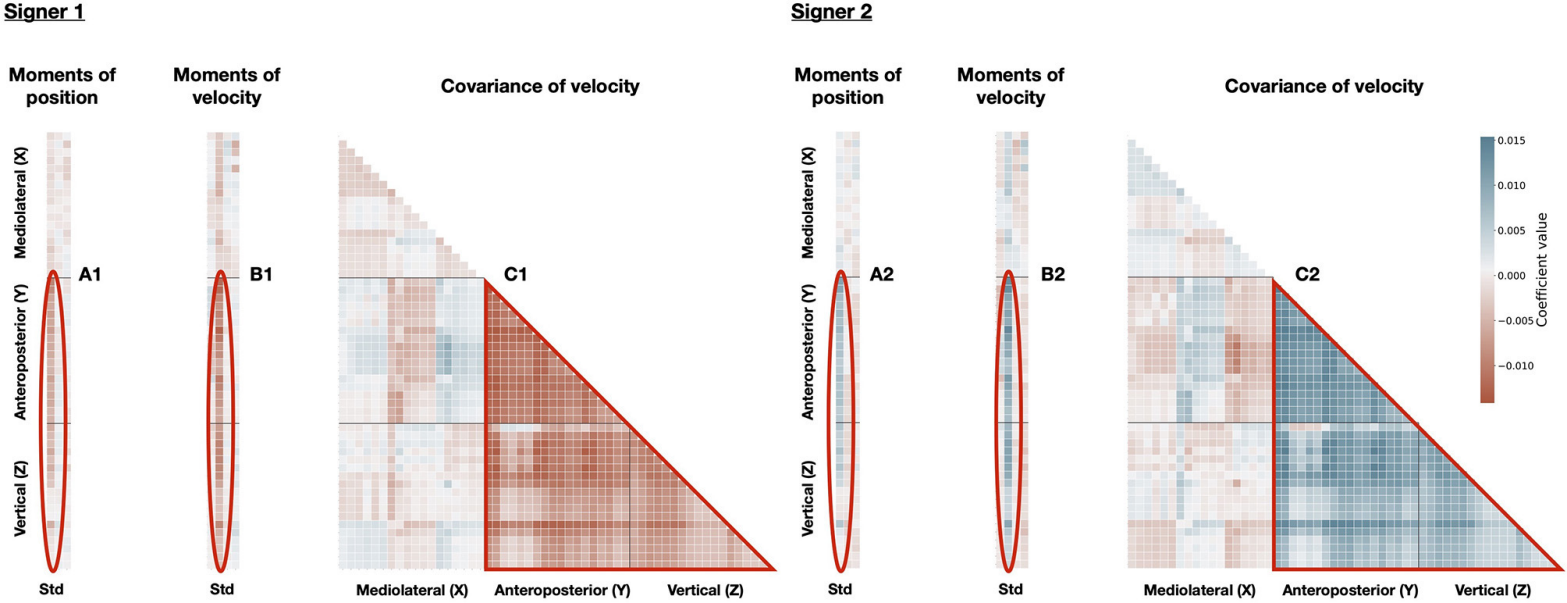
Classifier weights of PC1 for Signer 1 and Signer 2. Similar to Figure 6 for Signer 1 (left) and Signer 2 (right): moments (columns: std, skew, kurtosis) of position for all markers (rows), moments (columns: mean, std, skew, kurtosis) of velocity for all markers (rows), the covariance of velocity between markers (rows and columns). Markers are sorted from 1 to 19 as presented in the Methods along X, Y, and Z axes. Coefficients correspond to the logistic regression weights optimized for each signer. Blue represents positive weight values, while red represents negative ones. The three patterns of importance are highlighted: Std of position **(A1)** and velocity **(B1)** of all body markers for Signer 1; Std of position **(A2)** and velocity **(B2)** of all body markers for Signer 2; **(C1)** Covariance of velocity between all body markers along Y and Z axes for Signer 1; **(C2)** Covariance of velocity between all body markers along Y and Z axes for Signer 2.

These PCs, either combined or independently, can be used to discriminate between individual signers. For instance, Figure 7 displays the idiosyncratic statistical patterns (referred to as **d**_**n,k**_ in Equation 6) of some signers, along PC1. According to PC1, the movements of Signer 1 presented little relationship between anteroposterior and vertical axes (Figure 7C1), and low variation in position and velocity, along anteroposterior and vertical axes (Figures 7A1,B1). By contrast, Signer 2 characterized by a strong relationship between between anteroposterior and vertical axes (Figure 7C2) and high variation in position and velocity, along anteroposterior and vertical axes (Figures 7A2,B2). These discriminant PCs convey the motion signature of the identity of each signer, and they can be scrutinized in terms of the original statistics. These findings mean that identity can be inferred from simple statistics of kinematic features, with consistent accuracy.

## Discussion

The present study demonstrates that mocap data convey critical information to allow for robust identification of signers using machine learning, as previously shown for walking (Zhang and Troje, 2005) or dancing (Carlson et al., 2020). PCA followed by a linear classifier managed to correctly identify signers from the statistics of their movements recorded during the free description of pictures in spontaneous LSF. Even when deprived of structural information about the signers, the model reported 86.8% accuracy, over five times higher than the chance level. These results are consistent with prior findings on the human ability to identify individuals from walking (Cutting and Kozlowski, 1977; Troje et al., 2005; Westhoff and Troje, 2007) and dancing (Loula et al., 2005; Bläsing and Sauzet, 2018) movements. In particular, this is in line with recent behavioral evidence that humans can identify signers from PLDs of their movements in LSF (Bigand et al., 2020). Compared to the latter visual perception study, which measured the human ability to identify signers, the present study trained a machine learning model, which successfully identified signers from statistics of mocap data. Although with a feature analysis, Bigand et al. (2020) have shown that the size and shape of the body of signers may not have played a major in the accuracy of participants to identify, the machine learning approach taken in the present study allowed for the further determination of the specific features that allow for the identification.

The second outcome of the present study is that kinematics alone allow for robust identification of the signers. Removing size and shape information did not affect the performance of the model. Normalizing the mocap data with respect to the postures of signers led to a decline in identification accuracy. Nevertheless, the remaining identification accuracy was significantly above the chance level. The minor role of anthropometric differences in identifying individuals from their movements is consistent with prior behavioral studies on gait (Troje et al., 2005; Westhoff and Troje, 2007) and LSF (Bigand et al., 2020). Interestingly, the impact of the average posture of signers on the correct identification of the model was similar to the impact reported by Troje et al. (2005) on human observers, causing a decrease of about 10% of accuracy. The remaining ability of the model to identify signers without any of these structural cues confirms that kinematics alone are sufficient to achieve identification, as previously suggested by Troje et al. (2005) and Westhoff and Troje (2007) for walking.

Further analyses of the contribution of kinematic features to the identification of the model revealed identity-specific characteristics of the motion of signers. In general, discriminant PCs described specific kinematic statistics in all three dimensions. For instance, PC1, PC2, and PC4, which accounted for 54.9% of correct identification, were characterized by movements in the sagittal, frontal, and transverse planes, respectively. Previous findings have outlined critical features for gender classification of gait in the frontal plane, which are, therefore, best visible in frontal view (Mather and Murdoch, 1994; Troje, 2002). However, although Troje et al. (2005) have found an overall advantage for walker identification based on the frontal view, training on half-profile views allowed for higher performance when participants had to identify walkers from new viewpoints. Moreover, no overall advantage for the frontal view has been reported by Westhoff and Troje (2007), whose gait PLDs were totally deprived of structural information. Whereas, for now, the ability of human perceivers to identify signers have only been studied using frontal views (Bigand et al., 2020), half-profile and profile views may provide critical information, especially for kinematics. This observation is consistent with the recent machine learning model of dancer identification proposed by Carlson et al. (2020), which reported kinematic features of importance along all three dimensions.

Similar to Carlson et al. (2020), the discriminant PCs revealed distinct identity-specific patterns over sensors and dimensions. For instance, whereas PC1 reflected differences in the kinematics of all body markers along the anteroposterior axis, differences along the vertical axis concerned only the trunk (e.g., stomach, sternum, and shoulders) and head markers. PC1, PC2, and PC4 reported different contributions of each part of the bodies of signers, often distinguishing groups of markers such as head, trunk, or hand markers. We also noticed distinct contributions of the two hands, such as a lower impact of the left hand along the mediolateral axis in PC2 than the right hand. This may be due to the motion differences caused by the dominant hand of the signers, which was the right hand for all of them. Indeed, as with any other human movements, signers preferably use their dominant hand when signing, such as for pointing, fingerspelling (i.e., spelling out isolated words by producing letters with the hands) or one-handed signs, as this hand provides faster or more precise performance. Prior studies have highlighted inter-individual differences in the execution of principal movements (or eigenmovements) for skiing (Federolf et al., 2014), karate (Zago et al., 2017a) or pathological gait (Zago et al., 2017b). However, principal movements are based on frame-by-frame relations between gestures, while SL movements are hardly ever synchronized across examples and individuals. Hence, as previously pointed out by Tits (2018), we outlined here the advantage of using statistics as motion descriptors for identity, which is invariant to time and independent of semantic content.

The results of the present study suggest that signers have a kinematic signature, which is invariant to the semantic content of their movements in LSF. We were able to characterize this signature using 24 components extracted from PCA, leading to a 79.2% identification accuracy. Such a data-driven approach is particularly interesting in the case of identification as the discriminant features are mainly idiosyncratic and thus hard to define a priori for each individual. The other main advantage of PCA is its invertibility, which makes it possible to recompute statistics by projecting a linear combination of PCs back into the original space. These statistics could be manipulated (e.g. reducing, or exaggerating, the weight of the PCs of interest) while resynthesizing pre-recorded SL movements. To achieve this, we could develop algorithms similar to the ones used to synthesize sounds with matching statistics (McDermott et al., 2009; McDermott and Simoncelli, 2011; Norman-Haignere and McDermott, 2018). This approach would allow for the visualization of specific PCs, by exaggerating their weight in the combination of PCs, as previously shown for male and female gaits (Troje, 2002). Furthermore, being able to control identity-specific PCs in motion synthesis will provide promising perspectives toward anonymizing SL motion for virtual signers.

## Data Availability Statement

The raw data supporting the conclusions of this article will be made available by the authors, without undue reservation.

## Author Contributions

FB conceived the study, developed the computational models, and run the tests and analyzes and wrote the original draft of the manuscript. FB, EP, BB, and AB analyzed and interpreted the results. EP, BB, and AB supervised the project and provided critical revisions of the manuscript. All authors contributed to the paper and approved the submitted version.

## Conflict of Interest

The authors declare that the research was conducted in the absence of any commercial or financial relationships that could be construed as a potential conflict of interest.

## Footnotes

^1^https://media-pi.fr/

^2^Averaged over ORI, SI, SH and POST data, multinomial logistic regression reported a 92.7% (std=3.5%) accuracy, whereas an RBF kernel SVM reported a 85.8% (std=2.7%) accuracy.

## References

[B1] Atkinson A. P. Dittrich W. H. Gemmell A. J. Young A. W. (2004). Emotion perception from dynamic and static body expressions in point-light and full-light displays. Perception 33, 717–746. 10.1068/p5096 15330366

[B2] Baragchizadeh A. Jesudasen P. R. O'Toole A. J. (2020). Identification of unfamiliar people from point-light biological motion: a perceptual reevaluation. Vis. Cogn. 28, 513–522. 10.1080/13506285.2020.1834039

[B3] Benchiheub M.-E.-F. Berret B. Braffort A. (2016a). Collecting and analysing a motion-capture corpus of french sign language, in Workshop on the Representation and Processing of Sign Languages, Portoroz.

[B4] Benchiheub M.-E.-F. Braffort A. Berret B. Verrecchia C. (2016b). Mocap1. Available online at: https://www.ortolang.fr/market/item/mocap1. Limsi, distributed via ORTOLANG (Open Re-sources and TOols for LANGuage).

[B5] Bigand F. Prigent E. Braffort A. (2020). Person identification based on sign language motion: Insights from human perception and computational modeling, in Proceedings of the 7th International Conference on Movement and Computing (Jersey City), 1–7.

[B6] Bigand F. Prigent E. Braffort A. (2021). How fast is Sign Language? A reevaluation of the kinematic bandwidth using motion capture, in Proceedings of the 29th European Signal Processing Conference (Dublin).

[B7] Bläsing B. E. Sauzet O. (2018). My action, my self: Recognition of self-created but visually unfamiliar dance-like actions from point-light displays. Front. Psychol. 9:1909. 10.3389/fpsyg.2018.01909 30459668 PMC6232674

[B8] Blondel M. Boutet D. Catteau F. Vincent C. (2019). Signing amplitude and other prosodic cues in older signers: insights from motion capture from the signage corpus, in Corpora for Language and Aging Research (CLARe 4) (Helsinki).

[B9] Carlson E. Saari P. Burger B. Toiviainen P. (2020). Dance to your own drum: Identification of musical genre and individual dancer from motion capture using machine learning. J. New Music Res. 49, 162–177. 10.1080/09298215.2020.1711778

[B10] Catteau F. Blondel M. Vincent C. Guyot P. Boutet D. (2016). Variation prosodique et traduction poétique (lsf/français): que devient la prosodie lorsqu–elle change de canal?(prosodic variation and poetic translation (lsf/french): What happens to prosody with a channel change?)[in french], in Actes de la Conférence Conjointe JEP-TALN-RECITAL 2016. Vol. 1, (Paris: JEP), 750–758.

[B11] Cutting J. E. Kozlowski L. T. (1977). Recognizing friends by their walk: Gait perception without familiarity cues. Bull. Psychon. Soc. 9, 353–356. 10.3758/BF03337021

[B12] Federolf P. Reid R. Gilgien M. Haugen P. Smith G. (2014). The application of principal component analysis to quantify technique in sports. Scand. J. Med. Sci. Sports 24, 491–499. 10.1111/j.1600-0838.2012.01455.x 22436088

[B13] Gibet S. (2018). Building french sign language motion capture corpora for signing avatars, in Workshop on the Representation and Processing of Sign Languages: Involving the Language Community, LREC 2018 (Miyazaki).

[B14] Holt J. A. (1993). Stanford achievement test–8th edition: reading comprehension subgroup results. Am Ann Deaf 138, 172–175. 10.1353/aad.2012.0684

[B15] Johansson G. (1973). Visual perception of biological motion and a model for its analysis. Percept. Psychophys. 14, 201–211. 10.3758/BF03212378

[B16] Kozlowski L. T. Cutting J. E. (1977). Recognizing the sex of a walker from a dynamic point-light display. Percept. Psychophys. 21, 575–580. 10.3758/BF03198740

[B17] Latinus M. Belin P. (2011). Human voice perception. Curr. Biol. 21, R143–R145. 10.1016/j.cub.2010.12.033 21334289

[B18] Loula F. Prasad S. Harber K. Shiffrar M. (2005). Recognizing people from their movement. J. Exper. Psychol. Hum. Percept. Perform. 31:210. 10.1037/0096-1523.31.1.210 15709874

[B19] Lu P. Huenerfauth M. (2010). Collecting a motion-capture corpus of american sign language for data-driven generation research, in Proceedings of the NAACL HLT 2010 Workshop on Speech and Language Processing for Assistive Technologies (Los Angeles, CA), 89–97.

[B20] Lu P. Huenerfauth M. (2014). Collecting and evaluating the cuny asl corpus for research on american sign language animation. Comput. Speech Lang. 28, 812–831. 10.1016/j.csl.2013.10.004

[B21] Malaia E. Wilbur R. B. (2012). Kinematic signatures of telic and atelic events in asl predicates. Lang. Speech 55, 407–421. 10.1177/0023830911422201 23094321

[B22] Malaia E. Wilbur R. B. Milković M. (2013). Kinematic parameters of signed verbs. J. Speech Lang. Hear. Res. 56, 1677–1688. 10.1044/1092-4388(2013/12-0257) 23926292

[B23] Mather G. Murdoch L. (1994). Gender discrimination in biological motion displays based on dynamic cues. Proc. R. Soc. Lond. Ser. B Biol. Sci. 258, 273–279. 10.1098/rspb.1994.0173

[B24] McDermott J. H. Oxenham A. J. Simoncelli E. P. (2009). Sound texture synthesis via filter statistics, in 2009 IEEE Workshop on Applications of Signal Processing to Audio and Acoustics, (New Paltz, NY: IEEE), 297–300.

[B25] McDermott J. H. Schemitsch M. Simoncelli E. P. (2013). Summary statistics in auditory perception. Nat. Neurosci. 16, 493–498. 10.1038/nn.3347 23434915 PMC4143328

[B26] McDermott J. H. Simoncelli E. P. (2011). Sound texture perception via statistics of the auditory periphery: evidence from sound synthesis. Neuron 71, 926–940. 10.1016/j.neuron.2011.06.032 21903084 PMC4143345

[B27] Norman-Haignere S. V. McDermott J. H. (2018). Neural responses to natural and model-matched stimuli reveal distinct computations in primary and nonprimary auditory cortex. PLoS Biol. 16:e2005127. 10.1371/journal.pbio.2005127 30507943 PMC6292651

[B28] O'Toole A. J. Abdi H. Deffenbacher K. A. Valentin D. (1993). Low-dimensional representation of faces in higher dimensions of the face space. JOSA A 10, 405–411. 10.1364/JOSAA.10.00040528266751

[B29] Portilla J. Simoncelli E. P. (2000). A parametric texture model based on joint statistics of complex wavelet coefficients. Int. J. Comput. Vis. 40, 49–70. 10.1023/A:1026553619983

[B30] Sevdalis V. Keller P. E. (2009). Self-recognition in the perception of actions performed in synchrony with music. Ann. N.Y. Acad. Sci. 1169, 499–502. 10.1111/j.1749-6632.2009.04773.x 19673830

[B31] Tits M. (2018). Expert Gesture Analysis through Motion Capture using Statistical Modeling and Machine Learning (Ph.D. thesis), Ph.D. Dissertation.

[B32] Troje N. F. (2002). Decomposing biological motion: a framework for analysis and synthesis of human gait patterns. J. Vis. 2, 2–2. 10.1167/2.5.2 12678652

[B33] Troje N. F. Westhoff C. Lavrov M. (2005). Person identification from biological motion: Effects of structural and kinematic cues. Percept. Psychophys. 67, 667–675. 10.3758/BF03193523 16134460

[B34] Westhoff C. Troje N. F. (2007). Kinematic cues for person identification from biological motion. Percept. Psychophys. 69, 241–253. 10.3758/BF03193746 17557594

[B35] Zago M. Codari M. Iaia F. M. Sforza C. (2017a). Multi-segmental movements as a function of experience in karate. J. Sports Sci. 35, 1515–1522. 10.1080/02640414.2016.1223332 27560105

[B36] Zago M. Sforza C. Bona A. Cimolin V. Costici P. F. Condoluci C.. (2017b). How multi segmental patterns deviate in spastic diplegia from typical developed. Clin. Biomech. 48, 103–109. 10.1016/j.clinbiomech.2017.07.016 28806590

[B37] Zhang Z. Troje N. F. (2005). View-independent person identification from human gait. Neurocomputing 69, 250–256. 10.1016/j.neucom.2005.06.002

