# Supplementary figures and images for: Machine Learning of Motion Statistics Reveals the Kinematic Signature of the Identity of a Person in Sign Language
Source: Front Bioeng Biotechnol. 2021 Jul 22;9:710132. doi: 10.3389/fbioe.2021.710132 (PMC8342317; doi:10.3389/fbioe.2021.710132)

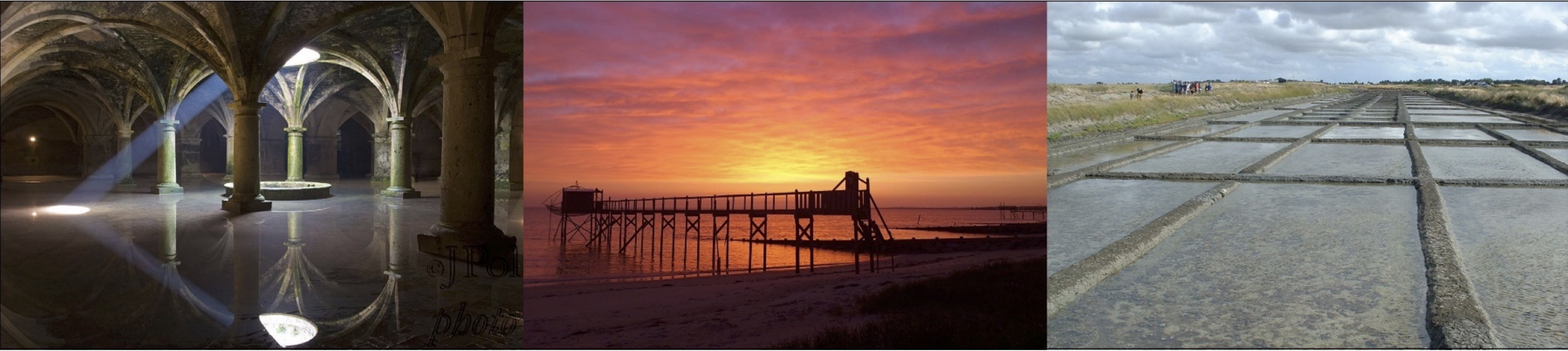

Supplement: Supplementary file 7 [file Image_1.JPEG]
